# 30.4 PRENATAL INFECTION AND LONG-TERM BRAIN PATHOLOGY: FROM PRECLINICAL MODELS TO MECHANISMS

**DOI:** 10.1093/schbul/sby014.125

**Published:** 2018-04-01

**Authors:** Juliet Richetto

**Affiliations:** University of Zurich

## Abstract

**Background:**

Prenatal exposure to infection is increasingly recognized to play an important etiological role in neuropsychiatric and neurological disorders with neurodevelopmental components, including schizophrenia, autism, bipolar disorder, and mental retardation. The adverse effects induced by prenatal infection may reflect an early entry into a deviant neurodevelopmental route, but the specificity of subsequent disease or symptoms is likely to be influenced by the genetic and environmental context in which the prenatal infectious process occurs. The epidemiological link between prenatal infection and increased risk of neurodevelopmental disorders also receives strong support from experimental work in animal models. These models are based on maternal gestational exposure to specific infectious agents such as influenza virus or immune activating agents such as the bacterial endotoxin lipopolysaccharide (LPS) or the viral mimic poly(I:C).

**Methods:**

Converging evidence form these models suggests that prenatal immune activation can negatively affect early foetal brain development and change the offspring’s neurodevelopmental trajectories, which in turn can lead to the emergence of behavioral and cognitive disturbances in later life. Modelling the human epidemiological association between prenatal infection and increased risk of neurodevelopmental disorders in animals has also greatly advanced our understanding of the underlying mechanisms. According to the prevailing view, cytokine-associated inflammatory events, together with downstream pathophysiological effects such as oxidative stress and (temporary) macronutrient and micronutrient deficiency, seem critical in mediating the post-acute effects of maternal infection on the foetal system.

**Results:**

Recent findings have further implicated epigenetic processes as possible molecular mechanisms translating the negative effects of prenatal immune activation on the offspring. Not only does prenatal immune activation cause long-lasting epigenetic modifications such as altered DNA methylation and miRNA expression, but it also causes a transgenerational transmission of behavioral and neuronal abnormalities without additional immune exposures.

**Discussion:**

Prenatal infection and associated developmental neuroinflammation may have a pathological role in shaping neurodevelopmental disease risk across generations.

